# RNA-seq analysis reveals key genes associated with seed germination of *Fritillaria taipaiensis* P.Y.Li by cold stratification

**DOI:** 10.3389/fpls.2022.1021572

**Published:** 2022-09-28

**Authors:** Qiu-Xiong Yang, Dan Chen, Yan Zhao, Xiao-Yu Zhang, Min Zhao, Rui Peng, Nian-Xi Sun, Timothy Charles Baldwin, Sheng-Chao Yang, Yan-Li Liang

**Affiliations:** ^1^ College of Agronomy & Biotechnology, Yunnan Agricultural University, Kunming, China; ^2^ Key Laboratory of Medicinal Plant Biology of Yunnan Province, Yunnan Agricultural Waseda University, Fengyuan, Kunming, China; ^3^ National & Local Joint Engineering Research Center on Germplasm Innovation & Utilization of Chinese Medicinal Materials in Southwestern China, Yunnan Agricultural University, Kunming, China; ^4^ Chongqing Academy of Chinese Materia Medica, Chongqing, China; ^5^ Faculty of Science and Engineering, University of Wolverhampton, Wolverhampton, United Kingdom

**Keywords:** seed dormancy, 4°C stratification, RNA-seq, phytohormone, WGCNA, *fritillaria taipaiensis* P.Y.Li

## Abstract

Seed dormancy is an adaptive strategy for environmental evolution. However, the molecular mechanism of the breaking of seed dormancy at cold temperatures is still unclear, and the genetic regulation of germination initiated by exposure to cold temperature requires further investigation. In the initial phase of the current study, the seed coat characteristics and embryo development of *Fritillaria taipaiensis* P.Y.Li at different temperatures (0°C, 4°C, 10°C & 25°C) was recorded. The results obtained demonstrated that embryo elongation and the dormancy-breaking was most significantly affected at 4°C. Subsequently, transcriptome analyses of seeds in different states of dormancy, at two stratification temperatures (4°C and 25°C) was performed, combined with weighted gene coexpression network analysis (WGCNA) and metabolomics, to explore the transcriptional regulation of seed germination in *F. taipaiensis* at the two selected stratification temperatures. The results showed that stratification at the colder temperature (4°C) induced an up-regulation of gene expression involved in gibberellic acid (GA) and auxin biosynthesis and the down-regulation of genes related to the abscisic acid (ABA) biosynthetic pathway. Thereby promoting embryo development and the stimulation of seed germination. Collectively, these data constitute a significant advance in our understanding of the role of cold temperatures in the regulation of seed germination in *F. taipaiensis* and also provide valuable transcriptomic data for seed dormancy for other non-model plant species.

## Introduction

Seed dormancy is an evolutionary adaption to habitat climate diversity, which results in a delay to seed germination (Willis et al., 2014; Finkelstein et al., 2008). It can also directly or indirectly impact the production of a wide variety of crops (Simsek et al., 2014). Therefore, seed dormancy plays a significant role in both plant ecology and agriculture. Seed dormancy is divided into four classes including: nondormancy (ND), physiological dormancy (PD), morphological dormancy (MD) and morphophysiological dormancy (MPD) (Baskin and Baskin, 2004). At present, research on seed dormancy has mainly focused on model plant species and seed physiological dormancy (PD) (Finkelstein and Reeves, 2018; Chahtane et al., 2017; Finch-Savage and Footitt, 2017) However, studies related to seed morphological dormancy (MD) are relatively uncommon (Walker et al., 2021). Our understanding of the molecular mechanism(s) of the breaking of seed dormancy at cold temperatures is even more limited.

*Fritillaria taipaiensis* P.Y.Li, a traditional Chinese medicinal plant, is important in Asia, both as a food crop and for its medicinal value. This species is a perennial alpine plant, indigenous to high-altitudes located in the mountainous regions of southwestern China. The process of seed dormancy in *F. taipaiensis* lasts for 200 days and is considered to be primarily due to morphophysiological dormancy (MPD). From our preliminary work, we found that cold temperatures may contribute to breaking the morphophysiological seed dormancy of this species. Therefore, we determined that the seed of *F. taipaiensis* provides good material for the investigation of seed germination initiated by exposure to cold temperatures.

It is widely known that both seed dormancy and germination are closely regulated by the crosstalk between the gene regulatory networks involved in the biosynthesis of the plant growth regulators ABA and GA (Gubler et al., 2005; Holdsworth et al., 2008; Graeber et al., 2012).

Expression of genes and transcription factors related to endogenous ABA biosynthesis, signal transduction and metabolism play a particularly important role in seed dormancy. The process whereby phytoxanthin is converted to xanthoxin, catalyzed by zeaxanthin cyclooxygenase (ZEP),9-cis-cyclooxygenase lyase (NCED), abscisic aldehyde oxidase (AAO) (Nambara and Marion-Poll, 2005; Arc et al., 2013) as a component of bioactive ABA synthesis *via* the carotenoid pathway is particularly important. Bioactive ABA can be glycosylated by UDP glucosyltransferase (UGT) to inactive ABA-GE (Liu et al., 2015). Conversely, β‐glucosidases encoded by *AtBg1* and *AtBg2* can convert ABA-GE into bioactive ABA (Lee et al., 2006; Xu et al., 2012). The signal transduction of ABA is also important. PYR/PYL/RCAR receptors can bind PP2C and inhibit its activity,and cause the release of SnRKs. The phosphorylation of SnRKs can activate the downstream signal cascade, ABA-activated SnRK2s phosphorylate Raptor and inhibit TOR activity, TOR and ABA signaling balance plant growth (Cutler et al., 2010; Hubbard et al., 2010; Wang et al., 2018). Transcription factors *ABI3* and *ABI5* also play a key role in the downstream regulation of the ABA signalling network (Piskurewicz et al., 2008; Barrero et al., 2010; Kanai et al., 2010; Penfield, 2017).

GA promotes seed germination by antagonizing and inhibiting ABA (Graeber et al., 2012). Previous studies showed that GAs anabolic pathway is comprehensive, and 136 types of GAs have been identified, four of which (GA_1_/GA_3_/GA_4_/GA_7_) have biological activity (Yamaguchi, 2008). GA_12_ is an important precursor, which can be catalyzed to produce bioactive GA by GA 20-oxidase (GA20ox) and GA 3-oxidase (GA3ox) (Sasaki et al., 2002; Appleford et al., 2006). Bioactive GAs can be deactivated by GA 2-oxidases (GA2oxs) (Sakamoto et al., 2001; Schomburg et al., 2003). The DELLA protein is also an important negative regulator involved in the GA signal transduction pathway, responding to environmental changes together with the GA receptor GIBBERELLIN INSENSITIVE DWARF 1 (GID1) (Harberd et al., 2009; Resentini et al., 2015). *DELAY OF GERMINATION-1*(*DOG1*) and *EM6* are the key regulators in seed primary dormancy (Nishimura et al., 2018; Carrillo-Barral et al., 2020).

Many other transcription factors and signalling networks also impact upon seed germination. *MFT* encodes MOTHER of FT and TFL1 protein, being activated and regulated by ABI3 and ABI5 during seed embryo development (Xi et al., 2010). *ZOUPI (ZOU)* is specifically expressed in endosperm tissue with INDUCER OF CBP EXPRESSION 1 (ICE1), which mediates the degradation of the surrounding endosperm and embryonic epidermal development during embryo growth. The ICE-CBF-COR module regulates the cold adaptation process in the model species *Arabidopsis thaliana*, in which ICE1 binds to MYC recognition sites in the CBF3 promoter to positively regulate CBF expression. *ICE1* expression is induced by low temperature, ICE1 mutation impairs chilling and freezing tolerance (Denay et al., 2014; MacGregor et al., 2019). GASSHO1 and GASSHO2 (GSO1 and GSO2) receptors are located on the embryonic surface, being responsible for signal communication between embryo and endosperm (Doll and Ingram, 2022). The expansin family of cell wall-associated proteins also plays an important role in promoting endosperm rupture, seed embryo growth and germination (Chen and Bradford, 2000; Chen et al., 2001; Yan et al., 2014; Muthusamy et al., 2020). Late embryonic development protein (LEA) is considered to be a marker during seed development and maturity (Shih and Hsing, 2008), participating in the response to various abiotic stresses (Wang et al., 2018; Czernik et al., 2020).

Although studies on seed dormancy are abundant in the literature, the progress on seed morphophysiological dormancy and seed dormancy breaking induced under cold temperatures requires further investigation. In the current study, the seed coat characteristics and embryo growth and development were recorded at different temperatures. In addition we also performed a transcriptome and endogenous hormone metabolome, with weighted gene coexpression network analysis (WGCNA), using the seeds of *F. taipaiensis* at nine different stages of dormancy. The main objectives of the study were (1) To identify differentially expressed genes and the response pathways of cold stratification. (2) To identify the key genes involved in the regulation of embryo elongation and seed germination under cold temperatures. (3) To explore the hub genes that link the cold temperature response to the regulation of embryo growth and development.

## Materials and methods

### Plant materials and growth conditions

Mature seeds of *F. taipaiensis* were surface sterilized with 20%(v/v) bleach for 5 min and then washed with sterile, distilled water. Turgid, shiny seeds were then selected for cultivation in petri dishes. Seeds were germinated on 2 layers of sterilized filter paper moistened under controlled conditions (0°C/4°C/10°C/25°C) for a period of two months, and each treatment was performed with three replicates (n=30, **Supplementary Table 3**). The rate of water absorption in the seed was assessed at various timepoints (0, 1, 3, 5, 10, 15, 20, 30,120, 240, 360, or 720min). The length of the embryo was counted each week, and the index of embryo elongation was calculated using the formula embryo rate (%) =seed embryo length/endosperm length. The seed coat surface morphology was observed by scanning electron microscopy (Yu et al., 2014).

### Sample collection and transcriptome sequencing

The mature seeds of *F. taipaiensis* used for transcriptome sequencing, were obtained from fresh seed capsules. The seeds were cultivated at 25°C in the controls, or at 4°C for the treatments.

**Figure 1:**
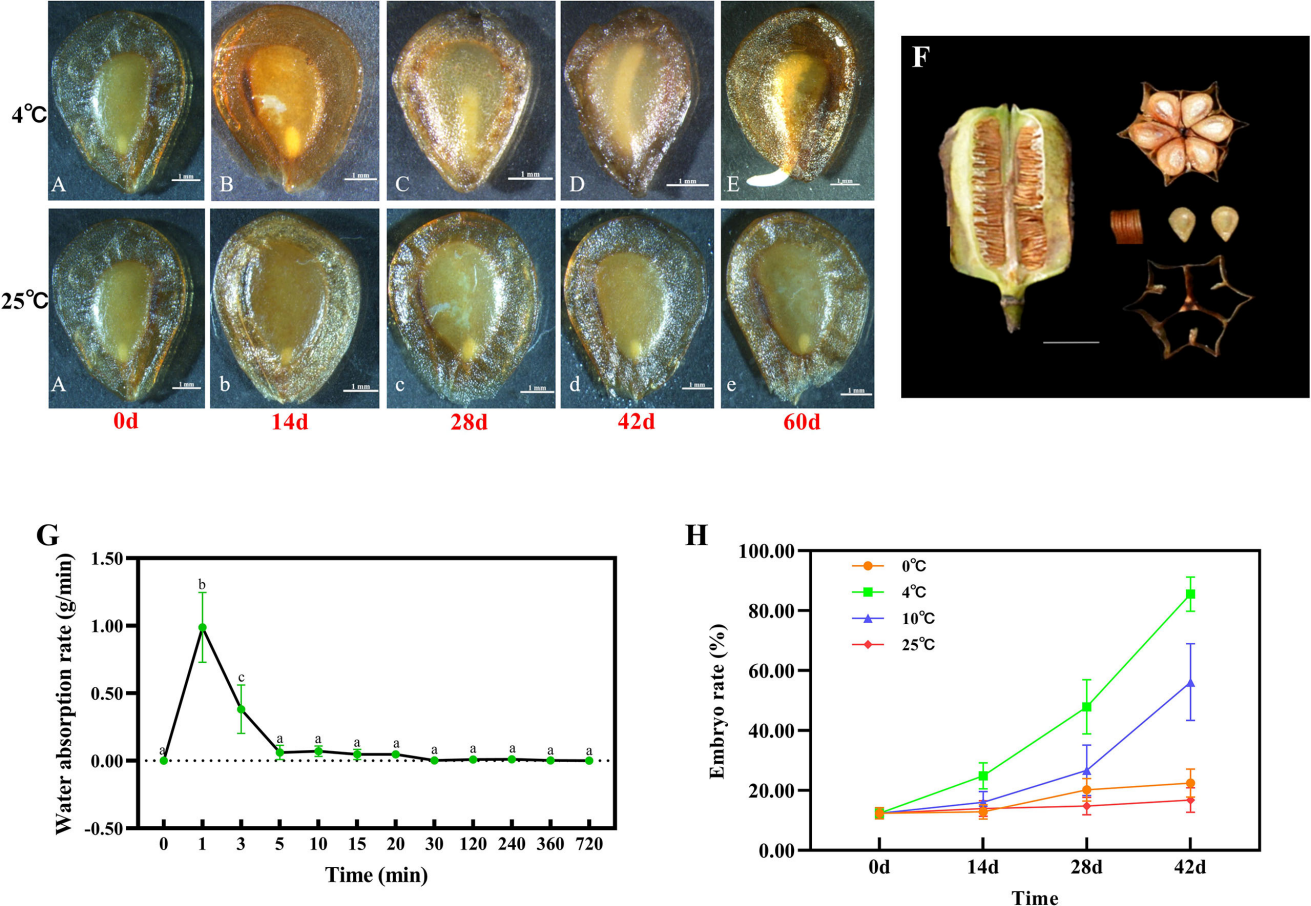
Phenotypic observation of the mature seed capsule and seed of *F. taipaiensis*. Seed embryo growth progression at 4°C **(A–E)** and 25°C **(b–e)**. The letters **(A–E)**, **(b–d)** and e represent 9 developmental stages of seed development, respectively (listed in **Supplementary Table 1**). Scale bar=1 mm. **(F)** Morphological and anatomical structure of mature seed capsule. Scale bar =1 cm. **(G)** The changes of seed water absorption rate. Values for each point were means ± SD (n = 5). Different letters indicate significant difference (P < 0.05). **(H)** The changes of embryo growth rate at different stratification temperatures of 0°C, 4°C, 10°C and 25°C. Values for each point were means ± SD (n = 30).

The embryos treated with stratification at 4°C were observed to elongate rapidly. The seeds stratified at 4°C were then subdivided in to five A, B, C, D & E samples. Sample A consisted of fresh seeds whilst samples B, C, D and E comprised of seeds cultivated at 4°C in which the embryo rate reached 25%, 50%, more than 90% and the radicle had broken through the seed coat by 1-3mm (**Figure 1**).

The seeds cultivated at 25°C were used as the control and samples of which were labelled b, c, d and e. The time of control sampling was consistent with the treatments and the length of embryo were almost unchanged. Three replicates of each of the 9 samples, containing a total of 27 samples were analysed (**Supplementary Table 1**). Each sample was snap frozen in liquid nitrogen and stored at -80°C. After mRNA extraction, cDNA library construction and transcriptome sequencing were performed using an Illumina HiSeq platform, by Metware sequencing services (http://www.metware.cn/).

### Transcriptome assembly and analysis

Trimmomatic v0.39 was used to filter the original sequence (Specific parameters: LEADING:20 TRAILING:20 SLIDINGWINDOW:4:20 MINLEN:50). RiboDetector v0.2.4 was utilized to remove rRNA sequences. *De novo* assembly was performed using Trinity v2.12.0 (k-mer:31, min_kmer_cov:2). The final unigenes were obtained from the cluster of assembled sequences using RapClust. The TransDecoder v5.5.0 was selected to predict the CDs region of unigenes, and BLAST v2.12.0 (The threshold of E-vaule = 1x10^-5^) was used to match the predicted CDs in the local KEGG, KOG, NR, NT, TrEMBL and Swissprot databases. The sequences were annotated to the Pfam database (version35.0) using the pfam-scan v1.6 program. GO and KO ortholog enrichment analyses were subsequently performed using interproscan v5.55-88.0 and kofam_scan v1.3.0. (The original data query accession numbers are: PRJNA874905, PRJNA874906, PRJNA874908, PRJNA874910, PRJNA874912, PRJNA874913, PRJNA874914, PRJNA874917, PRJNA874920. Fasta data in **Supplementary Material 1**, BUSCO 5.3.2,embryophyta: C: 92.0%[S:84.4%,D:7.6%], F:4.3%, M:3.7%, n: 1614).

### RT-qPCR

Total RNA was extracted using a HiPure HP Plant RNA Mini Kit (Guangzhou Meiji Biotechnology Co., Ltd.). First-strand cDNA was synthesized from RevertAidTM First strand cDNA Synthesis (TransGen Biotech, Beijing, China). The PCR reactions were carried out on QuanstudioTM 5 Real-Time PCR Instrument (Thermo Fisher Scientific, Inc.), using ChamQ Universal SYBR qPCR Master Mix (Vazyme Biotech Co., Ltd.). A ubiquitin gene was selected as the internal reference, and primers were designed by Primer3 (https://primer3.ut.ee/). The amplification reaction mixture consisted of 10 µL of 2× ChamQ Universal SYBR qPCR Master Mix, 0.5 µL each of 10 mM forward and reverse primers, 1 µL of cDNA template, and 6 µL of ddH_2_O in a final volume to 18 µL. The qPCR reaction conditions were: pre-denaturation: 95°C for 30 sec, cyclic reaction: 95°C for 10 sec, 60°C for 30 sec, 40 cycles, lysis curve reaction: 95°C 15 sec, 60°C 1 mine, 9°C 15 sec. Relative expression values were obtained using the 2^-ΔΔCt^ method (Livak and Schmittgen, 2001). The primers used for fluorescence quantification are shown in the **Supplementary Table 2**.

### Determination of endogenous hormones

The seeds of 5 developmental stages (A/B/C/D/E) were used to determine the endogenous hormone content. ABA, IAA, and cytokinin were detected by MetWare (http://www.metware.cn/). Three replicates of each assay were performed.

### WGCNA analysis

A phenotype weighted coexpression network was constructed in the R-package WGCNA with 27 samples and 9 phenotypes (Langfelder and Horvath, 2008), all differentially expressed genes were submitted to WGCNA, adjusting a batch using limma’s removeBatchEffect (Ritchie et al., 2015), and the establishment of 9 module features (A/B/C/D/E/b/c/d/e). The soft threshold was calculated using the pickSoftThreshold function. The modules were obtained using the automatic network construction function blockwiseModules. The eigenvalues of each module were calculated and used to test the correlation of each trait. P values < 0.05 was considered significant. K_ME_ was used to express the connectivity of genes in specific modules. The network connectivity between nodes was analysed using 11 algorithms of the Cytoscape plugin cytoHubba (version 3.8.2) (Chin et al., 2014). The unigenes with high connectivity were selected as hub genes based on the results of Maximal Clique Centrality (MCC) analysis.

## Results

### Cold ambient air temperatures (4°C) may significantly promote embryo elongation in *F. taipaiensis*

Seeds of *F. taipaiensis* are flat, heart-shaped or obovate, and the observed 1000 grain weight in the current study was 4.45 ± 0.17g. The seed coat was shown to be highly permeable, and its epidermal cells were loosely arranged (**Supplementary Figure 1**). The water absorption rate of dry seeds reached 0.98 ± 0.25g/min, while the water was absorbed into seeds for 1-3min. Seeds were observed to become fully imbibed within 30min (**Figure 1G**).

The immature embryo was embedded in an abundant living endosperm, that occupied most of the seed’s volume (**Figure 1A**). The length of the embryo accounted for 12.38% of the size of endosperm (**Figure 1H**, **Supplementary Table 3**). These seeds exhibit the characteristic features of morphological dormancy.

In order to investigate the effect of temperature upon seed germination, seeds were grown under the different temperature regimes (0, 4, 10 and 25°C), and the phenotype of germination was observed (**Figure 1H**). The results showed that the relatively cold temperature (4°C) had the most significant effect on embryo elongation and dormancy-breaking. The embryo rate reached about 25% after cold stratification for 14 days at 4°C (**Figure 1B**). The embryo rate accounted for 50% and more than 85.5% of the endosperm (mature embryo) after 28 days and 42 days successively (**Figures 1C, D**). The seeds were then placed in warmer environmental conditions (15-20°C, 7-10 days) to complete their germination (**Figure 1E**, **Supplementary Figure 2**). The growth rate of embryos pretreated at 10°C was slower than those stratified at 4°C. The embryo rate accounted for 16%, 26% and 56% of the endosperm after 14 days, 28 days and 42 days (**Figure 1H**). The embryos treated at 0°C or 25°C showed no significant change at the same time. These data suggest that the best stratification temperature (of those examined) at which to break the seed dormancy of *F. taipaiensis* is 4°C.

### RNA-sequencing analysis

To understand how cold temperature promotes seed germination, three biological replicates were selected and a total of 27 seed samples were used for transcriptomic analysis. The samples (named A, B, C, D, E, b, c, d and e) are shown in **Figure 1**. Approximately 177.09 Gb clean data were generated. An average of 93% raw reads had a quality score of Q30 (**Supplementary Table 4, Sheet 1**). A total of 67,784 unigenes were obtained and 61,574 unigenes could be matched to KEGG, NR, Swiss-Prot, KOG and TrEMBL databases (**Supplementary Table 4, Sheet 2**). The pearson correlation analysis of the RNA-seq data showed that the repeatability of the data was good (**Supplementary Figure 3**). PCA analysis indicated that high PC1 scores exhibited 17.94% of the trait variance for different time points, and PC2 explained 12.45% of the total variance for genotypes (**Supplementary Figure 4**).

**Figure 2:**
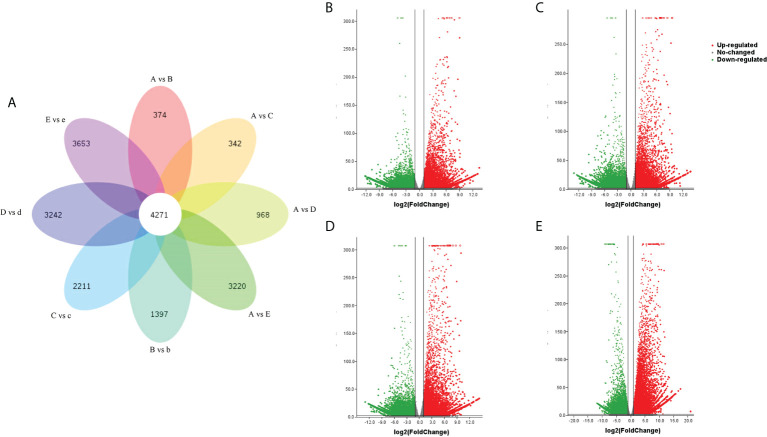
Transcriptomic analysis of the DEG distribution in the nine seed samples. **(A)** Venn diagram representation of the stage-specific DEGs distribution in the seed samples. A vs B, A vs C, A vs D, A vs E, B vs b, C vs c, D vs d and E vs e represent the DEGs in each pairwise comparison, respectively. **(B–E)** Differentially expressed gene volcano map of cold treatment for 14 d, 28 d, 42 d or germination. **(B–E)** were represent A vs B, A vs C, A vs D, and A vs E, respectively. Differentially expressed genes were: expression difference foldlog2FoldChange> 1 and significance p-value < 0.05.

To identify changes in our transcriptome data that occurred during the seed germination under cold treatment, the TPM (Transcripts Per Million) values were used to measure genes or transcripts expression levels. Differentially expressed genes (DEGs) (expression difference fold | log2FoldChange | > 1 and significance p-value < 0.05) were identified among samples A, B, C, D and E. Each was compared with samples b, c, d and e. The results showed that 374, 342, 968 and 3,220 DEGs were identified between A vs B, A vs C, A vs D, A vs E, respectively. There were 1,397, 2,211, 3,242 and 3,653 DEGs between B vs b, C vs c, D vs d, E vs e, respectively. It was found that the number of DEGs were enriched during the germination time points. 4,271 unigenes exhibited conserved expression at the 9 developmental stages (**Figure 2A**).

### Functional enrichment analysis

**Figure 3:**
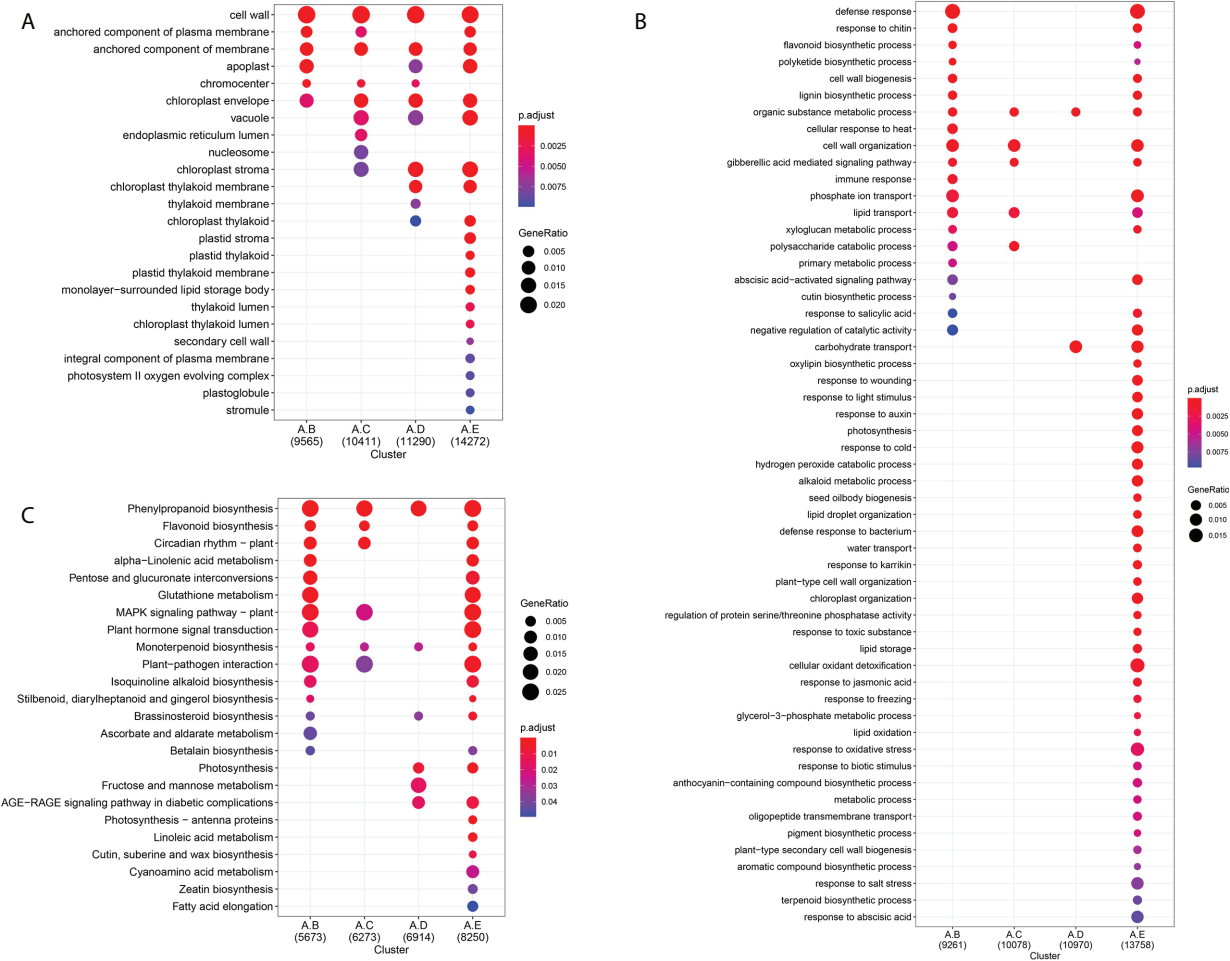
GO and KEGG enrichment analysis of DEGs related to seed germination by 4°C treatment for 0d, 14 d, 28 d, 42 d or germination. **(A)** GO analysis of the terms enriched in the DEGs among to the 4 comparisons (A vs B, A vs C, A vs D, A vs E). The top 24 terms of most enriched GO terms belong to cellular composition. padj < 0.01. **(B)** The top 55 terms of most enriched GO terms belong to biology process. padj < 0.01. **(C)** KEGG enrichment pathways. The left side of each figure represents the enriched function terms. The dot size indicates the number of GeneRatio. padj < 0.05.

The GO enrichment analysis was used to classify the function of the unigenes, which were differentially expressed during seed germination under cold stratification. A total of 37,327 unigenes were distributed into the different known GO terms. Of them, 32,246 unigenes were annotated in GO-MF terms, 9,565 unigenes in GO-CC terms, 29,095 unigenes in GO-BP terms (**Figure 3A, B**, **Supplementary Figure 5**). Among the “biological process” category, the most significantly enriched terms were “response to oxidative stress”, “cell wall organization”, “response to abscisic acid”, “carbohydrate transport”, “response to cold”, “response to auxin”. The top enriched GO terms in “molecular functions” were related to “hydrolase activity, hydrolyzing O-glycosyl compounds”, “oxidoreductase activity”, “transferase activity”, “UDP-glycosyltransferase activity”, “glutathione transferase activity”, “signaling receptor activity”, “abscisic acid binding”, “pectate lyase activity”. Moreover, GO analysis showed that more significant enrichment in developmental stage E than in stage A at different functional modules.

To identify the functions of DEGs related to seed germination at 4°C, KEGG pathway enrichment analysis was performed for 14 days, 28days and 48days germination at 4°C stratification, compared to fresh seeds. The top significant enriched pathways were “Phenylpropanoid biosynthesis”, “Plant hormone signal transduction”, “MAPK signaling pathway-plant”, “Fructose and mannose metabolism”, “Pentose and glucuronate interconversions”, “Linoleic acid metabolism” The number of unigenes enriched in these pathways showed an upward trend in developmental stages A-E (**Figure 3C**). Therefore, based on the results of KEGG, the MAPK signaling, hormone signaling, and fructose and mannose metabolism related pathways were selected as being the most important pathways related to plants’ perception and adaptation to cold temperature, and which are thereby implicated in the control of seed germination of *F. taipaiensis* seeds at 4°C.

### Identification of DEGs involved in endogenous hormone regulation pathway

**Figure 4:**
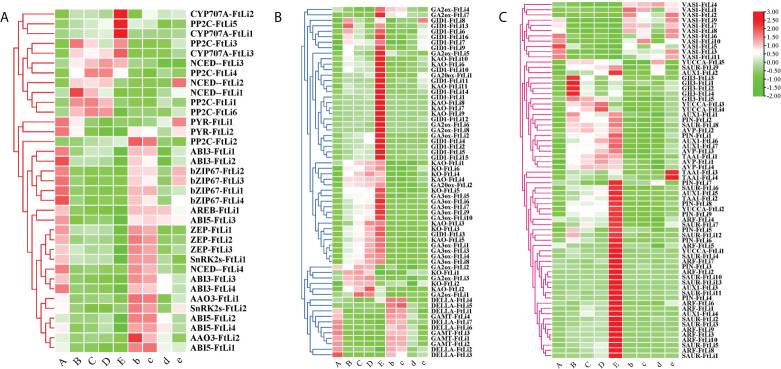
Heatmaps of the expression of ABA - **(A)**, GA- **(B)**, and auxin pathway **(C)** related genes between the treatments and controls. (A-E) represents the treatments and “b”, “c”, “d” “e” represents the controls, respectively. Red and green represent up- and downregulated transcripts, respectively. All genes are listed in detail in **Supplementary Table 5**.

To elucidate the genetic regulation of the ABA pathway in the maintenance of seed dormancy and the breaking of seed dormancy, the DEGs were filtered and compared to those previously reported to be involved in ABA biosynthetic, transport, and signaling pathways. Heatmap analysis of the DEGs involved in ABA biosynthesis, revealed that their expression was inhibited at 4°C compared to 25°C, such as *ZEP (ZEP-FtLi.1, ZEP-FtLi.2, ZEP-FtLi.3)* and *NCED(NCED-FtLi.4)* (**Figure 4A**, **Supplementary Table 5**). Furthermore, the expression of the *PYR* receptors (*PYR-FtLi.1, PYR-FtLi.2*), kinases *SnRK2s* (SnRK2s-FtLi.1, SnRK2s-FtLi.2), transcription factors *ABI3(ABI3-FtLi.1, ABI3-FtLi.2, ABI3-FtLi.3, ABI3-FtLi.4)*, and transcription factors *bZIP67*(*bZIP67-FtLi.1, bZIP67-FtLi.2, bZIP67-FtLi.3, bZIP67-FtLi.4*) were also significantly reduced. It was also found that the expression of *ABI5 (ABI5-FtLi.1, ABI5-FtLi.2, ABI5-FtLi.4)* was induced at 25°C. The *PP2C*(*PP2C-FtLi.4*) and *CYP707A* (*CYP707A-FtLi.1, CYP707A-FtLi.2, CYP707A-FtLi.3)* genes that are responsible for inactivating/inhibiting ABA biosynthesis were significantly upregulated in developmental stages A to E stages, but most significantly in sample E.

Many of the DEGs were genes involved in the *de novo* biosynthesis of active gibberellin (**Figure 4B**), such as *KAO* (*KAO-FtLi1, KAO-FtLi3, KAO-FtLi4, KAO-FtLi5*), *KO* (*KO-FtLi3, KO-FtLi4, KO-FtLi5, KO-FtLi6*), *GA3ox* (*GA3ox-FtLi1, GA3ox-FtLi2, GA3ox-FtLi3, GA3ox-FtLi4, GA3ox-FtLi8*) and *GA20ox* (*GA20ox-FtLi1, GA20ox-FtLi2)*. These genes were significantly expressed at 4°C compared to 25°C. In accordance with this biosynthetic process, the expression of the *GID1* (*GID1-FtLi6, GID1-FtLi7, GID1-FtLi8, GID1-FtLi9, GID1-FtLi16*) family of receptor genes was also upregulated. Stratification at 4°C was shown to inhibit the expression of the repressor gene *DELLA* (*DELLA-FtLi1, DELLA-FtLi2* and *DELLA-FtLi3*) and the inactivation gene *GAMT* (*GAMT-FtLi1, GAMT-FtLi2, GAMT-FtLi3, GAMT-FtLi4*).

We filtered DEGs for those previously reported to be involved in auxin biosynthesis, transport, and signaling pathways (**Figure 4C**). The auxin biosynthesis genes *YUCCA* (*YUCCA-FtLi.3*, *YUCCA-FtLi.4*), *TAA1*(*TAA1-FtLi.1*) and auxin efflux carrier genes *PIN* (*PIN-FtLi.2*), the influx transporter genes *AUX1*(*AUX1-FtLi.1, AUX1-FtLi.2, AUX1-FtLi.6, AUX1-FtLi.7*) showed the largest upregulation under cold stratification. *ARF* (*ARF-FtLi.1, ARF-FtLi.3, ARF-FtLi.5, ARF-FtLi.6*) and *SAUR* (*SAUR-FtLi.8, SAUR-FtLi.9, SAUR-FtLi.6*) were DEGs related to auxin signaling pathways, which were all shown to be highly expressed when the radicles ruptured the seed coat in the 4°C stratification treatments. The expression of the *GH3*(*GH3-FtLi.1, GH3-FtLi.2, GH3-FtLi.3, GH3-FtLi.4, GH3-FtLi.5*) gene family was shown to be significantly increased in stage B. The aminotransferase VAS1, negatively regulates IAA biosynthesis. Cold stratification significantly inhibited *VAS1-FtLi.6, VAS1-FtLi.7, VAS1-FtLi.8, VAS1-FtLi.9, VAS1-FtLi.10* expression.

ICE1 plays a positive regulatory role in the response to cold stress. Cold stratification upregulated the expression of *ICE1*, including *ICE1-FtLi.4, ICE1-FtLi.7, ICE1-FtLi.10, ICE1-FtLi.13* and *ICE1-FtLi.19*. By contrast, 4°C pretreatment inhibited the expression of several dormancy maintenance genes, including *DOG1* (*DOG1-FtLi3, DOG1-FtLi4, DOG1-FtLi5, DOG1-FtLi6, DOG1-FtLi7 and DOG1-FtLi8*)*, EM6*(*EM6-FtLi1, EM6-FtLi2* and *EM6-FtLi3*) and *MFT* (*MFT-FtLi2*). In the current study, 24 genes were identified encoding for expansin proteins that play a pivotal role in primary cell wall loosening and cell expansion. Throughout seed embryo maturation, *EXPA-FtLi14* was highly expressed. The proteins (LEA-FtLi) play a critical role during seed maturation, and the genes that encode for it (*LEA-FtLi2, LEA-FtLi4, LEA-FtLi5, LEA-FtLi6, LEA-FtLi8*) showed significant upregulation in stage B. Furthermore, we identified several genes involved in carbohydrate metabolism, including α-amylase (AMY), β-amylase (BAM) and mannan endo-1,4-beta-mannosidase (MAN). The enzyme genes *AMY-FtLi.3, AMY-FtLi.5, BAM-FtLi.7, BAM-FtLi.8, MAN-FtLi.4, MAN-FtLi.5* were highly expressed from stage B to stage E (**Supplementary Figure 6**).

### Identification of key genes and modules during seed germination by WGCNA

**Figure 5:**
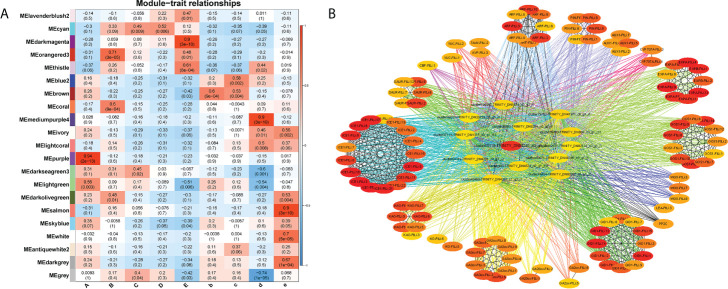
WGCNA of DEGs between the 9 development stages. **(A)** Module–trait association of the 9 sample groups (A, B, C, D, E, b, c, d and e). Each row corresponds to a module labeled with a colour. Each column corresponds to one sample group. **(B)** Interaction network analysis of the MEdarkmagenta module. The top 120 hub genes were used to build interaction network diagram in the MEdarkmagenta module. Dots represent hub genes. All details are listed in **Supplementary Tables 9**, **10**.

A weighted gene co-expression network analysis (WGCNA) can construct gene interaction networks, as well as identify gene modules and hub genes within them. We performed a WGCNA using our RNA-seq data to identify genes that link the cold response to plant growth and development. For the purpose of reducing noise, we only included genes that were differentially expressed across at least one comparison (A vs. B, A vs. C, A vs. D, A vs. E, B vs. b, C vs. c, D vs. d or E vs. e). For the analysis of a network topology, 14 was set as the soft-thresholding power (**Supplementary Figures 7A, B**). Based upon pairwise correlation analysis of gene expression, two main branches could be identified among the 21 merged coexpression modules (**Supplementary Figure 7C**). According to the module-trait correlation analysis for the 27 samples, 4 modules (*R* > 0.90) were significant (purple, darkmagenta, mediumpurple4 and salmon), and which were significantly correlated with samples A, E, d, e. These modules had gene numbers that ranged from 1,426 (salmon) to 6,620 (darkmagenta) (**Figure 5A**, **Supplementary Table 6**), and the K_ME_ (eigengenes connectivity) value of each gene was calculated. We found that stage A was tightly associated with the MEpurple module (*R* = 0.94 and *p* = 2×10^-13^), and stage E with the MEdarkmagenta module (*R* = 0.90 and *p* = 3×10^-10^). Next, we annotated the unigenes in these two modules with GO and KEGG, and found that only unigenes in the medarkmagenta module were enriched by KEGG. The unigenes were shown to be enriched in the biosynthetic pathways such as starch and sucrose metabolism, riboflavin metabolism, isoflavonoid biosynthesis, fatty acid elongation, cutin, suberine and wax biosynthesis, flavonoid biosynthesis, carotenoid biosynthesis and photosynthesis (**Supplementary Data Figure 10**, *p.adjust*<0.04). Therefore, we suspected that the hub genes might be related to the carbohydrate metabolism and the synthesis and transport of endogenous hormones. Therefore, the hub genes could play a connective role to link seed germination and resultant seedling morphogenesis.

Seed dormancy and germination are closely linked to *ICE1, ARF, KAO, GA3ox, GID1, PP2C, YUC, SAUR, PIN, WOX, LEA, Expansin*, and *GOS1.* The MEdarkmagenta module was used to mine the genes connected with *ICE1, ARF, KAO, GA3ox, GID1, PP2C, YUC, SAUR, PIN, WOX, LEA, Expansin, GOS1* were mined (**Supplementary Table 7**). Twelve algorithms were used to evaluate the connectedness of these genes (**Supplementary Table 8**). Gene networks were constructed using MCC score rankings. Based on the node scores, the top 120 hub genes were identified in the MEdarkmagenta module (**Figure 5B**, **Supplementary Table 9**). In the top 120 hub genes, 107 unigenes were related to endogenous hormones and plant growth and development. The role of the remaining 13 unigenes is unclear. We clarified the functions of hub genes by homologous sequence alignment (TAIR, https://www.arabidopsis.org/Blast/index.jsp), these genes encoded some important proteins involved in endogenous hormone signalling (TTL3, ATGSTU17), transcription factors (bHLH and AHL24), carbohydrate and energy metabolism (AT4G00905, NDPK3 and ARFA1B), the cell cycle, plant cell wall metabolism (PATATIN-LIKE PROTEIN 2 and AT5G02640), chloroplast biosynthesis (PHOTOSYSTEM II BY), and lignin biosynthesis (ATBCB) (**Supplementary Table 10**). Cold stratification progressively increased the expression levels of these 13 hub genes until the highest level was reached at germination stage E. Whereas at 25°C the expression of these genes was almost unchanged (**Supplementary Figures 8**).

### RNA-seq validation using qRT-PCR

**Figure 6:**
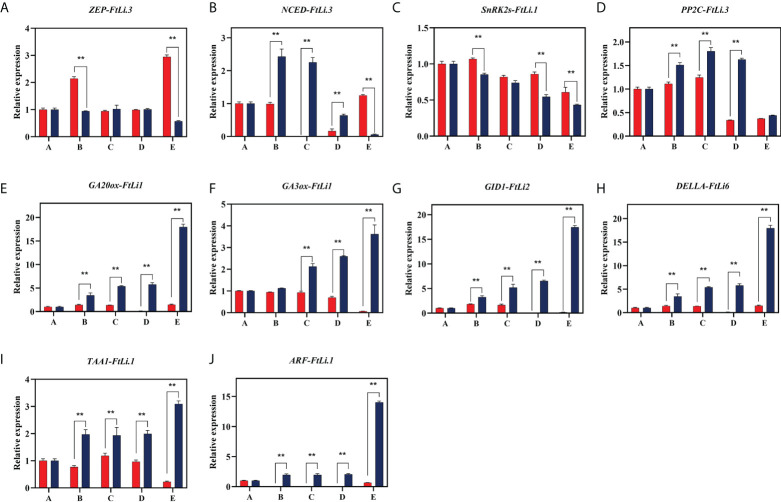
The RT-qPCR validation of the expression levels of genes related to endogenous hormones present in *F. taipaiensis*. The values presented are the means + SD of three biological replicates. The transcript IDs and the primers of each gene are listed in **Supplementary Tables 2**, **12**. A(ZEP,zeaxanthin cyclooxygenase), B(NCED,9-cis-cyclooxygenase lyase), C(SnRK2s,Snf1‐related protein kinases 2), D(PP2C,PP2C phosphatases), E(GA20ox,GA 20-oxidase), F(GA3ox,GA 3-oxidase), G(GID1,GIBBERELLIN INSENSITIVE DWARF 1), H(DELLA, DELLA protein), I(TAA1,tryptophan aminotransferase 1), J(ARF, AUXIN RESPONSE FACTOR). ** indicate significant difference (P < 0.01).

To assess the reliability of the differentially expressed transcripts, 10 candidate unigenes were selected and analyzed using qRT-PCR (primers listed in **Supplementary Table 2**). These genes are known to function in seed dormancy and germination processes, including GAs pathways (*GA20ox*, *GA3ox*, *GID1*), and ABA pathways (*ZEP*, *NCED*, *SnRK2_S_*, *PP2C*), as well as auxin biosynthesis (*TAA1*) and response (*ARF*) (**Figure 6**). These changes in gene expression were consistent with the RNA-seq data. As a result of the cold treatment, *ZEP-FtLi.3*, *NCED-FtLi.1* and *SnRK2s-FtLi.1* gene expression was suppressed, but *PP2C-FtLi.1* expression was promoted. *GA20ox-FtLi.1, GA3ox-FtLi.*1, *GID1-FtLi.1*, *TAA1-FtLi.1* and *ARF-FtLi.1* were also positively promoted by 4°C stratification (**Figures 6E–G**). Furthermore, our transcriptome sequencing results were shown to be reliable.

### The verification of transcriptome analysis data with regards to the endogenous hormone regulation pathway

**Figure 7:**
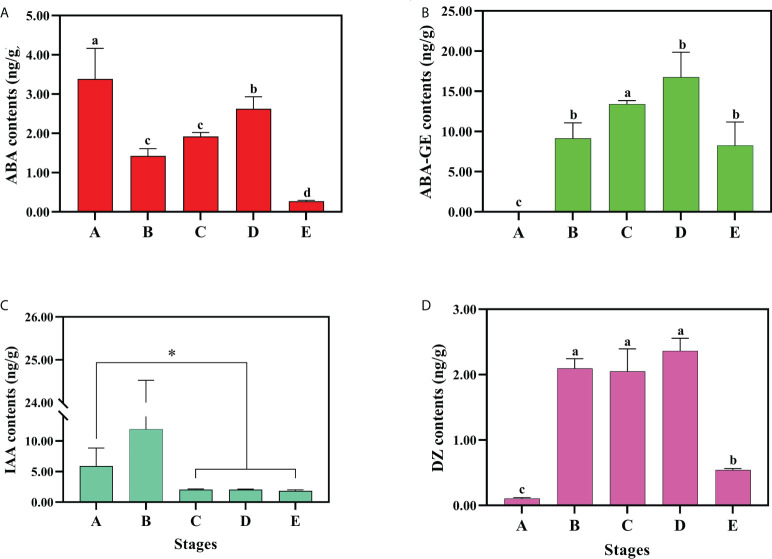
The endogenous hormone content There were contents changes of bioactive or inactive **(A)** ABA, **(B)** ABA-GE, **(C)** IAA and **(D)** DZ. Three biological replicates for each sample and analyzed by ANOVA. Values are reported as means ± SE (n = 3). * indicate significant difference (P < 0.05).

According to the transcriptome data, we found that endogenous hormones regulate seed dormancy and germination. To verify the role of the genes of endogenous hormone regulation pathway, we measured the hormone concentrations in developmental stages A, B, C, D and E (**Figure 7**, **Supplementary Table 11**). The bioactive ABA concentrations were shown to sharply decline as a consequence of the stratification at 4°C, with the highest concentration recorded at developmental stage A (3.38 ± 0.77ng/g) and the lowest concentration at stage E (0.27 ± 0.02ng/g). In stages B and C, the concentrations of bioactive ABA were as low as 1.42 ± 0.18ng/g and 1.92 ± 0.09ng/g, respectively, suggesting that cold treatment indeed can promote ABA biodegradation or biological inactivation. It is of interest to note that the content of bioactive ABA increased to 2.62 ± 0.30ng/g at developmental stage D. In contrast to ABA, the ABA-GE did not show any significant change in content (**Figures 7A, B**). Meanwhile, the concentrations of bioactive auxin and cytokinin, that were observed to significantly increase in stage B showed the opposite trend with ABA. As the embryo began to elongate (developmental stage B), the content of IAA markedly increased, but decreased in the following stages (**Figure 7C**). The level of IAA-amino acid conjugates were significantly changed, the content of IAA-Trp, IAA-Asp and IAA-Glu decreased from stage A to E, but IAA-Glc was shown to increase (**Supplementary Figure 9**). Among the four bioactive forms of cytokinin, the content of DZ reached 2.0ng/g, and maintained a steady high level throughout developmental stages B, C and D, then fell back down to 0.54 ± 0.02ng/g in stage E (**Figure 7C**). These results suggest that DZ plays an important role in the process of embryo development.

## Discussion

Globally speaking seed dormancy and germination are regulated by a diverse range of external environmental factors (Baskin and Baskin, 2004; Finkelstein et al., 2008; Rajjou et al., 2012). Temperature is a key environmental factor that affects the breaking of seed dormancy and subsequent seed germination (Simon et al., 1976; Yang et al., 2019). The majority of recent research on seed dormancy has mainly focused on the model species Arabidopsis, rice, *Brachypodium* (Hao et al., 2021), *Apium graveolens* (Walker et al., 2021), wheat (Gao et al., 2012), *Euphorbia esula* L. (Foley et al., 2010).

It is known that wheat seed can be released from a physiologically dormant state by after-ripening. Comparative transcriptomic analysis dormant (D) and post mature (AR) seeds in a dry state showed that the response elements of ABA increased during wheat dormancy, and that there was a linkage between wheat dormancy and the sensitivity of seeds to ABA. Studies on the regulation of *Euphorbia esula* seed germination under the alternate temperature of day and night also showed that genes related to abscisic acid signalling were the key regulators of this physiological process.

However, in recent years few reports on the seed dormancy of other plant species have been published. In the current study, we examined the role of cold temperature stratification (at 4°C) in breaking the dormancy of *F. taipaiensis* seed. Transcriptomic analysis was carried out to determine differentially expressed pathways and genes, qRT-PCR was performed to confirm their reliability, endogenous hormones were quantified, and WGCNA was used to investigate hub genes. Our results provide a comprehensive analysis of the regulatory mechanisms that promote seed germination of *F. taipaiensis* under cold treatment.

### Cold temperature is required to break dormancy of *F. taipaiensis* seed

Seed germination is a key and complex process in the plant life cycle, the optimum temperature for which is species specific. Cold stratification is widely used to break dormancy and trigger germination in many species (Kim et al., 2019). Generally, cold stratification to break seed dormancy is most effective between 0 and 10°C (Chen et al., 2020). However, it is still largely unknown as to how cold stratification breaks the dormancy in these species.

*F. taipaiensis* is a typical alpine plant, which requires precise seed dormancy adjustment to ensure seedling safety. According to Baskin, the type of seed dormancy exhibited by *F. taipaiensis* belongs to the morphological and physiological comprehensive dormancy grouping. Phenotypic analysis has shown that embryos from plants of this species treated at 10 °C develop quite slowly, and those treated at 0°C or 25°C did not develop hardly at all, but seeds placed at 4°C quickly broke their morphological dormancy (**Figure 1**). In this study we have demonstrated that cold temperature of 4°C plays an important role in the seed germination of *F. taipaiensis.*

To further investigate the molecular mechanism of cold regulation the seed germination of *F. taipaiensis*, RNA-Seq was performed. Altogether 177.09 Gb clean data was obtained, the clean reads were between 6.16-7.56G, base of Q30 percentage was 93% (**Supplementary Table 4**), and 61,574 unigenes were annotated. The enrichment analysis of GO and KEGG pathways showed that the most significant enriched pathways were “Phenylpropanoid biosynthesis”, “Plant hormone signal transduction”, “MAPK signaling pathway-plant”, “Fructose and mannose metabolism”, “Pentose and glucuronate interconversions”, “Linoleic acid metabolism” (**Figure 3C**). Therefore, it was proposed that these pathways were significantly related to seed dormancy breaking in this species.

### Hormonal regulation contributes to cold temperature germination of *F. taipaiensis* seed

A great deal of research has been performed related to how various genes function in the biosynthesis and signal transduction of hormones during seed dormancy and germination in both Arabidopsis and rice (Nonogaki, 2014).

From these studies it has been demonstrated that seed dormancy is determined not only by endogenous hormones ABA and GA, but that it is also indirectly influenced by cold temperatures (Finkelstein et al., 2008; Yamaguchi, 2008; Rajjou et al., 2012; Arc et al., 2013; Lv et al., 2021). In the study presented, several genes related to ABA, GA and auxin were shown to be differentially expressed in the process of seed dormancy breaking under 4°C stratification.

The endogenous hormone ABA is an important chemical compound in the induction and maintenance of seed dormancy (Kucera et al., 2005; Nonogaki, 2017). The expression of key enzymes for ABA synthesis *ZEP-FtLi.1* and *NCED-FtLi.4* were shown to be inhibited at 4°C compared to 25°C (**Figure 4A**, **Figures 6**, **7**), as well as the changing trend of ABA content during seed germination. These results revealed that dynamic changes of ABA levels played an important role in the process of seed germination at the colder temperature (4°C).

ABA signaling networks are initiated and operate through the expression of receptor PYR/PYL genes (Di et al., 2018). According to SK Yadav, PYL genes (*AtPYL/2/4/5/8/9*) and *AtPYL6/13* have different functions, the former promotes seed germination, whereas the latter has the opposite effect (Yadav et al., 2020). In our study, the expression of *PYR-FtLi.1/2* was inhibited by cold temperature stratification, indicating its positive role in the seed dormancy of *F. taipaiensis*. The expression pattern of protein phosphatases (PPS) can also respond to stress (He et al., 2019), which is important for cell homeostasis (Singh et al., 2016). The SnRK2s and PP2C kinases play opposite roles in the ABA signaling pathway. The decreased expression of *SnRK2s-FtLi.1* and the increased expression of *PP2C-FtLi.4* showed that cold temperature inhibited positive ABA signal transduction. ABI5 is a key transcription factor that regulates ABA signaling and inhibits seed germination (Piskurewicz et al., 2008). The expression of *ABI5 (ABI5-FtLi.1, ABI5-FtLi.2, ABI5-FtLi.4)* was induced by higher temperatures in our experiment. These results strongly suggest that cold temperature played a role in the germination of *F. taipaiensis* by inhibiting ABA signaling pathway.

Gibberellins play a role in the promotion of germination (Finkelstein et al., 2008). GA bound to GID1, inhibites DELLA activity and thus promotes seed germination (Voegele et al., 2011; Hauvermale et al., 2015). In the current study, cold stratification of the seed was shown to positively regulate the GA signaling pathway by causing expressions of *GID1-FtLi6/7/8/9* and inhibiting *DELLA-FtLi1/2/3* under 4°C stratification (**Figure 4B**, **Figure 6H**). The GA 20-oxidase (GA20ox), GA 3-oxidase (GA3ox) proteins are the key rate-limiting enzymes for bioactive gibberellin synthesis (Sasaki et al., 2002; Appleford et al., 2006), and their expression was shown to be induced by cold, and remained low or even absent at 25°C stratification (**Figure 4B**). These results indicate a major role of GAs in the cold germination of *F. taipaiensis* seed.

Previous studies have shown that auxin is involved in seed germination (Mohamed et al., 2022). IAA is the main bioactive form of auxin in plants, while *TAA* and *YUCCA* encode the key enzymes involved in the auxin biosynthetic pathway (Stepanova et al., 2008; Won et al., 2011). Both *TAA1-FtLi.1* and *YUCCA-FtLi.3/4* were shown to be upregulated during the breaking of seed dormancy in *F. taipaiensis.* Moreover, as the embryo elongated at stage B (**Figure 7C**), the content of IAA was also observed to increase significantly, thus confirming the transcriptome results. Both *PIN* and *AUX1* were upregulated, encoding auxin efflux carriers and influx transporters under cold stratification. ARF and SAUR are known to play important roles in IAA signal transduction (Park et al., 2007; Pierdonati et al., 2019). *ARF* and *SAUR* expression levels were significantly improved during seed germination. GH3 can convert bioactive IAA to IAA-amino acid (Aoi et al., 2020), and the *GH3-FtLi.1/2/3* gene family were shown to be highly expressed at developmental stage B. The expression model of *GH3* was consistent with the variation of IAA-Glc (**Figure 4C**, **Supplementary Figure 9E**). In our data, cold temperature stratification at 4oC was demonstrated to significantly inhibit the expression of *VAS1*, and thus promote auxin catabolism. These results strongly indicate that auxin is an essential regulator of cold germination in seeds of this species.

### Hub gene mining of integrated signals

The gene networks MEpurple and MEdarkmagent were identified as being related to seed germination and dormancy, respectively. *HEC3* (AT5G09750) was one of the hub genes in the MEdarkmagen module which can regulate tissue development (Riechmann et al., 2000; Gremski et al., 2007). It can be observed from our data that *HEC3* plays a central role in the regulation of seed germination (**Figure 5B**). The hub genes *ARFA1B* and *TTL3* are involved in auxin and brassinosteroid mediated signaling pathways (Ceserani et al., 2009). The hub gene *PSBY* was also identified, which is involved in the formation of the photosynthetic system (Sydow et al., 2016). The expression pattern of all the hub genes was similar, in that the levels of expression increased gradually with cold perception, reaching the highest level at the seed germination stage (**Supplementary Figure 8**). Therefore, it is clear that these hub genes play a key role in the breaking of seed dormancy in *F. taipaiensis* seed at cold temperatures, and that further studies on their molecular function(s) in this process are required.

## Conclusions

**Figure 8:**
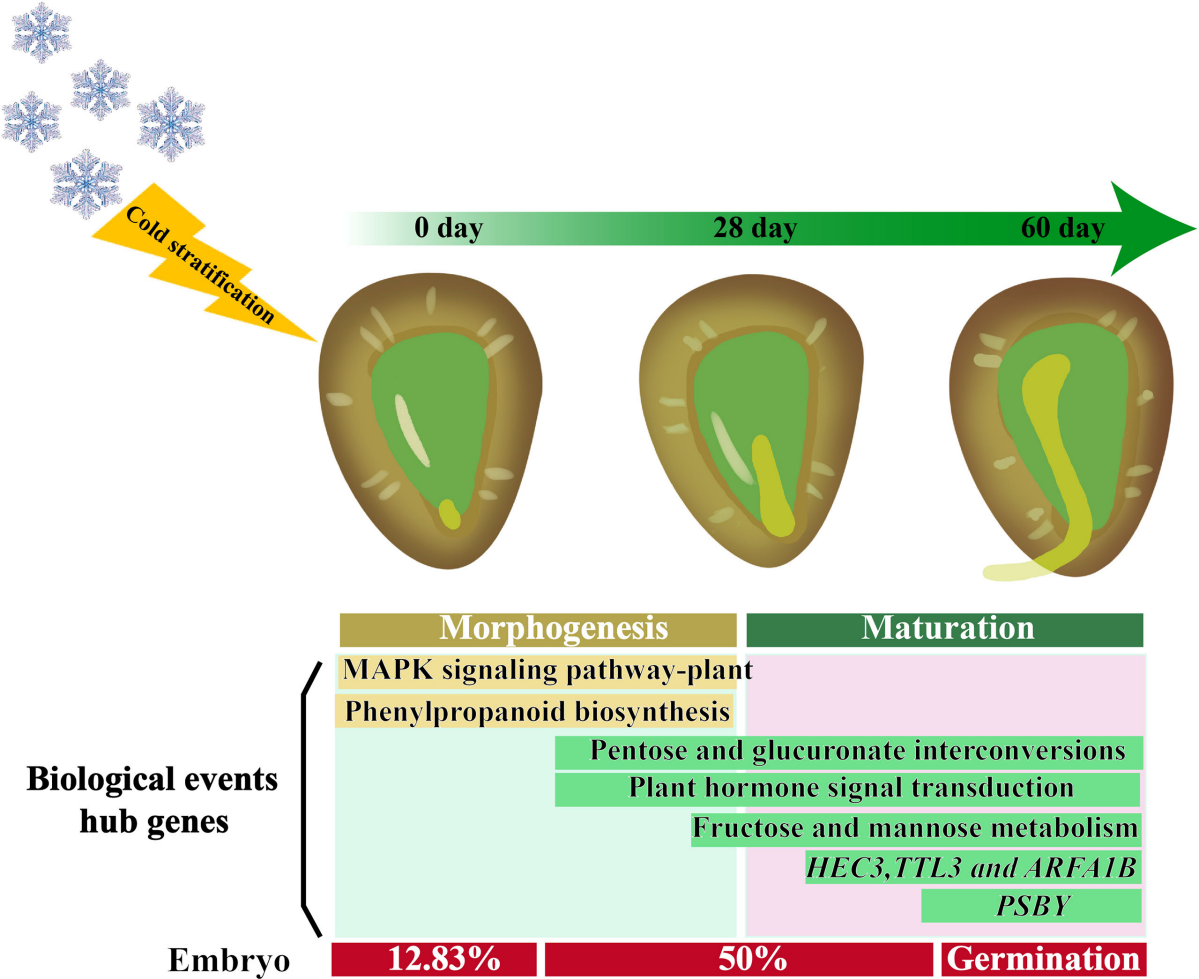
Proposed model of dormancy-breaking of *F. taipaiensis* seeds by cold stratification. The dormancy breaking process of *F. taipaiensis* seeds is defined as: When seeds ‘sensed’ cold stimulus, the pathways “MAPK signaling pathway of plant” and “Phenylpropanoid biosynthesis” in the seeds are quickly responded, and the pathways such as “Pentose and glucuronate interconversions”, “Plant hormone signal transduction” and “Fructose and mannose metabolism” are activated successively and throughout the process of dormancy breaking. Besides, the hub genes *HEC3*, *TTL3*, *ARFA1B* and *PSBY* seems to play an important role in the seed germination progress.

The study presented provides an in-depth analysis of the transcriptomes of seeds of *F. taipaiensis* with different dormancy levels. We propose a model of the dormancy-breaking mechanism in *F. taipaiensis* seeds treated with cold temperature stratification which explains some key aspects the molecular mechanism of cold temperature germination in this species (**Figure 8**). The analyses of key metabolic pathways presented and of the differentially expressed genes, revealed that cold temperature stratification may up regulate GA and auxin pathways, inhibit the ABA pathway, and promote the embryo development and subsequently stimulate seed germination. Based on WGCNA, the hub genes in the core regulatory position were screened. In future experiments, we will investigate the function of these critical genes and identify their roles in regulating embryo expansion. Our research also provides valuable transcriptomic data for seed dormancy of non-model plants, which may enrich our understanding the mechanism of plant embryo expansion and seed germination.

## Data availability statement

The datasets presented in this study can be found in online repositories. The names of the repository/repositories and accession number(s) can be found in the article/**Supplementary Material**.

## Author contributions

Y-LL and S-CY directed the whole process of this project. They also assisted in the writing of the manuscript. Q-XY participated in the whole project, analyzed experimental data, and helped write the paper. DC conducted the most of experiments. YZ helped with the conception and writing of the manuscript. X-YZ, MZ, RP, N-XS and TB provided help and advice on the experimental design and data analysis and TB proofread the final draft of the manuscript. All authors contributed to the article and approved the submitted version.

## Funding

This research was supported by the National Natural Science Foundation of China (31971543) and by the Major Special Science and Technology Project of Yunnan Province (202102AA310031,202101BD070001-008, 202102AA310045, 202005AC160040).

## Conflict of interest

The authors declare that the research was conducted in the absence of any commercial or financial relationships that could be construed as a potential conflict of interest.

## Publisher’s note

All claims expressed in this article are solely those of the authors and do not necessarily represent those of their affiliated organizations, or those of the publisher, the editors and the reviewers. Any product that may be evaluated in this article, or claim that may be made by its manufacturer, is not guaranteed or endorsed by the publisher.
