# Supplementary material for: RNA-seq analysis reveals key genes associated with seed germination of Fritillaria taipaiensis P.Y.Li by cold stratification
Source: Front Plant Sci. 2022 Sep 28;13:1021572. doi: 10.3389/fpls.2022.1021572 (PMC9555243; doi:10.3389/fpls.2022.1021572)
Supplement: Supplementary file 2 [file Table_1.docx]

Supplementary Materials

# 1 Supplementary Tables

**Table S1:** The materials of *F. taipaiensis* seed used in RNA-seq.

**Table S2:** List of primers used in the RT-qPCR validation.

**Table S3:** Stratification progression of embryo growth at different temperatures.

**Table S4:** The RNA-seq data of *F. taipaiensis* seed.

**Table S5:** Genes involved in endogenous hormones and seed dormancy signaling-pathways.

**Table S6:** The details of unigenes in these four modules in the WGCNA.

**Table S7:** The connectedness between candidate unigenes in the MEdarkmagenta-module networks.

**Table S8:** Identification of hub genes in the MEdarkmagenta modules using 12 algorithms in CytoHubba.

**Table S9:** The details of top 120 hub genes in MCC scores by cytohubba.

**Table S10:** Functional and expressions patterns on the 13 hub genes of the MEdarkmagenta-module.

**Table S11:** The contents of endogenous hormones at 5 stages.

**Table S12:** Relative expression levels of several genes related to endogenous hormones using RT-qPCR analysis.

# 2 Supplementary Figures

**FIGURE S1 The SEM of seed coat microstructure. (A)** and **(B)**shows the surface of seed coat. Scale bar=1mm. **(C)** seed surface pattern. Scale bar =200um. **(D)** micropyle region. Scale bar =200um.

**FIGURE S2 Germination rates of matured seeds at 15 ℃.** An analysis of the change in the germination rate of matured seed after stratification at 4℃. Different letters indicate significant differences among the time series (p <0.05). Lines represent SD of 30 replicates. Germination rate was scored at 8 time points (T:1 d, 3 d, 5 d, 7 d, 9 d, 11d, 13d and 15d).

**FIGURE S3 Pearson correlation analysis of transcriptome data between different samples.** The Pearson correlation coefficients between different samples were calculated by the cor function in R package and presented as heatmap using pheatmap software.

**FIGURE S4 Principal component analysis (PCA) with 27 samples.** Each group contains three biological replications.

**FIGURE S5** **GO terms analysis enriched in the DEGs common to the 4 comparisons (A vs B, A vs C, A vs D, A vs E).** The top 28 terms of most enriched GO terms belong to molecular function. padj < 0.01.

**FIGURE S6** **Heat maps of DEGs involved in the pathways of (A) seed dormancy and cold signaling, (B) embryo development, and (C) energy metabolism.** Red and green represent up- and down-regulated transcripts, respectively. All genes are listed in detail in Supplementary Table S5.

**FIGURE S7 Determination of soft thresholding and hierarchical cluster tree in the WGCNA.** **(A)** Soft threshold (power) screening. R^2^=0.9. **(B)** Analysis of the soft threshold and mean connectivity. **(C)** Hierarchical cluster tree showing the coexpression modules identified in the WGCNA. 21 modules make up the major tree branches.

**FIGURE S8 The heat map of the 13 hub genes.** Red and green represent up- and down-regulated transcripts, respectively. All genes are listed in detail in Supplementary Table S10.

**FIGURE S9** **The contents of inactive endogenous auxin. (A)** and **(B)** are TRP and IAN, respectively. **(C), (D), (E)** and **(F)** represent IAA-Trp, IAA-Asp, IAA-Glu and IAA-Glc, respectively. Three biological replicates for each sample. Three biological replicates data were analyzed by ANOVA. Values are reported as means ± SE (n = 3). TRP, L-tryptophan; IAN, 3-Indoleacetonitrile; IAA-Trp, Indole-3-acetyl-L-tryptophan; IAA-Asp, Indole-3-acetyl-L-aspartic acid; IAA-Glu, Indole-3-acetyl glutamic acid; IAA-Glc, 1-O-indol-3-ylacetylglucose.

**FIGURE S10** **KEGG enrichment analysis of DEGs in the medarkmagenta module.** The left side of figure represents the enriched function terms. The dot size indicates the number of GeneRatio. padj < 0.04.
