# Supplementary figures and images for: RNA-seq analysis reveals key genes associated with seed germination of Fritillaria taipaiensis P.Y.Li by cold stratification
Source: Front Plant Sci. 2022 Sep 28;13:1021572. doi: 10.3389/fpls.2022.1021572 (PMC9555243; doi:10.3389/fpls.2022.1021572)

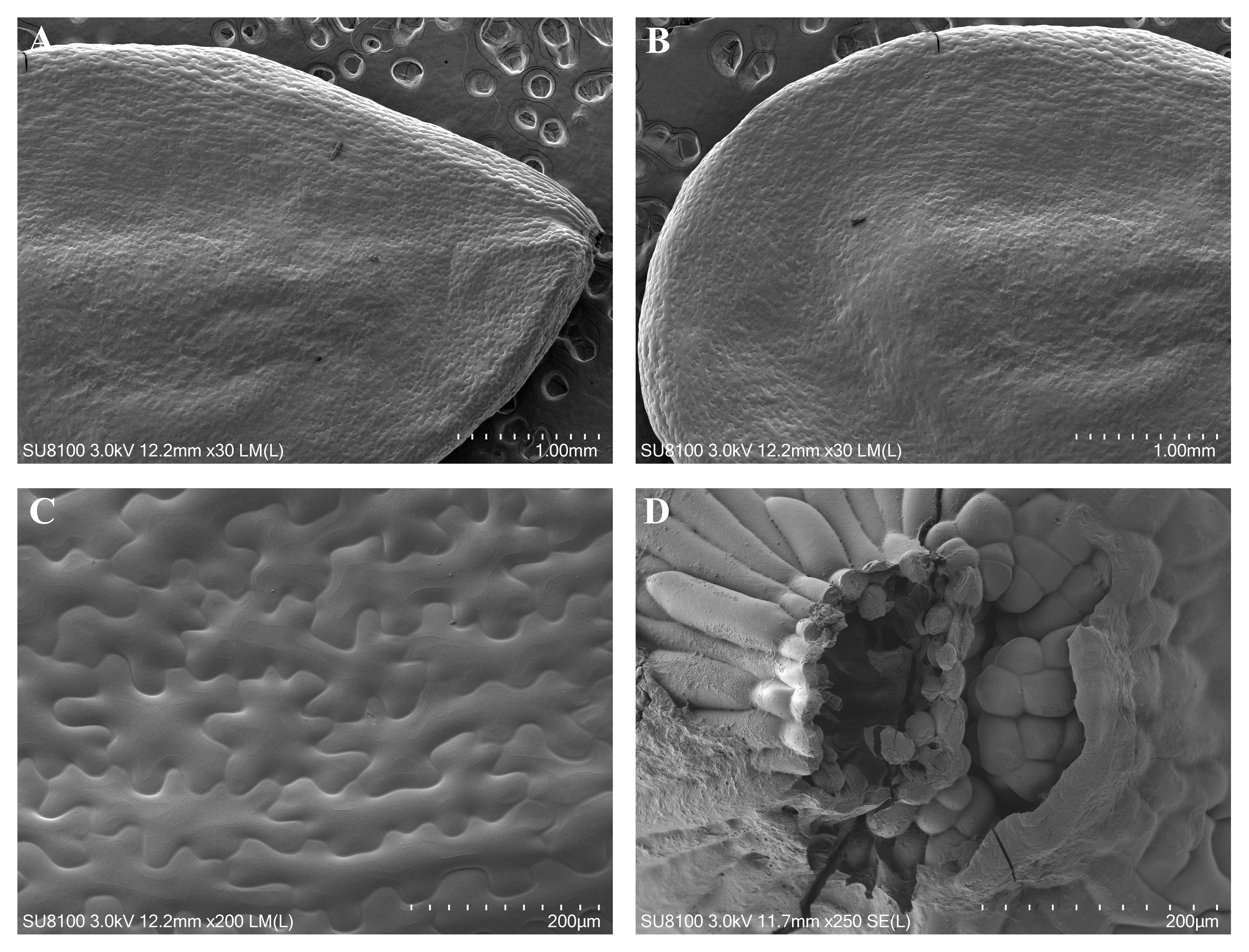

Supplement: Supplementary file 15 [file Image_1.jpeg]

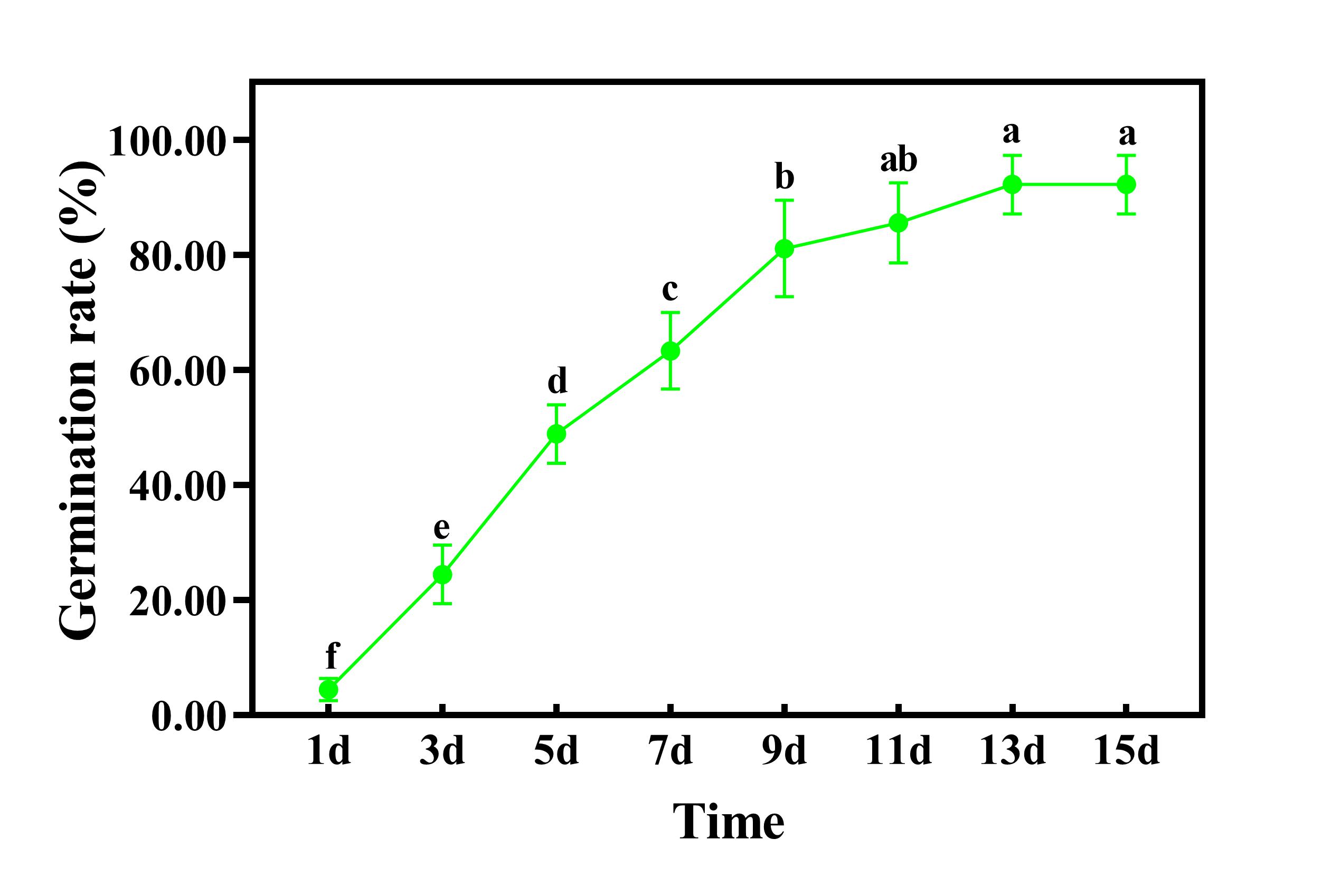

Supplement: Supplementary file 16 [file Image_2.jpeg]

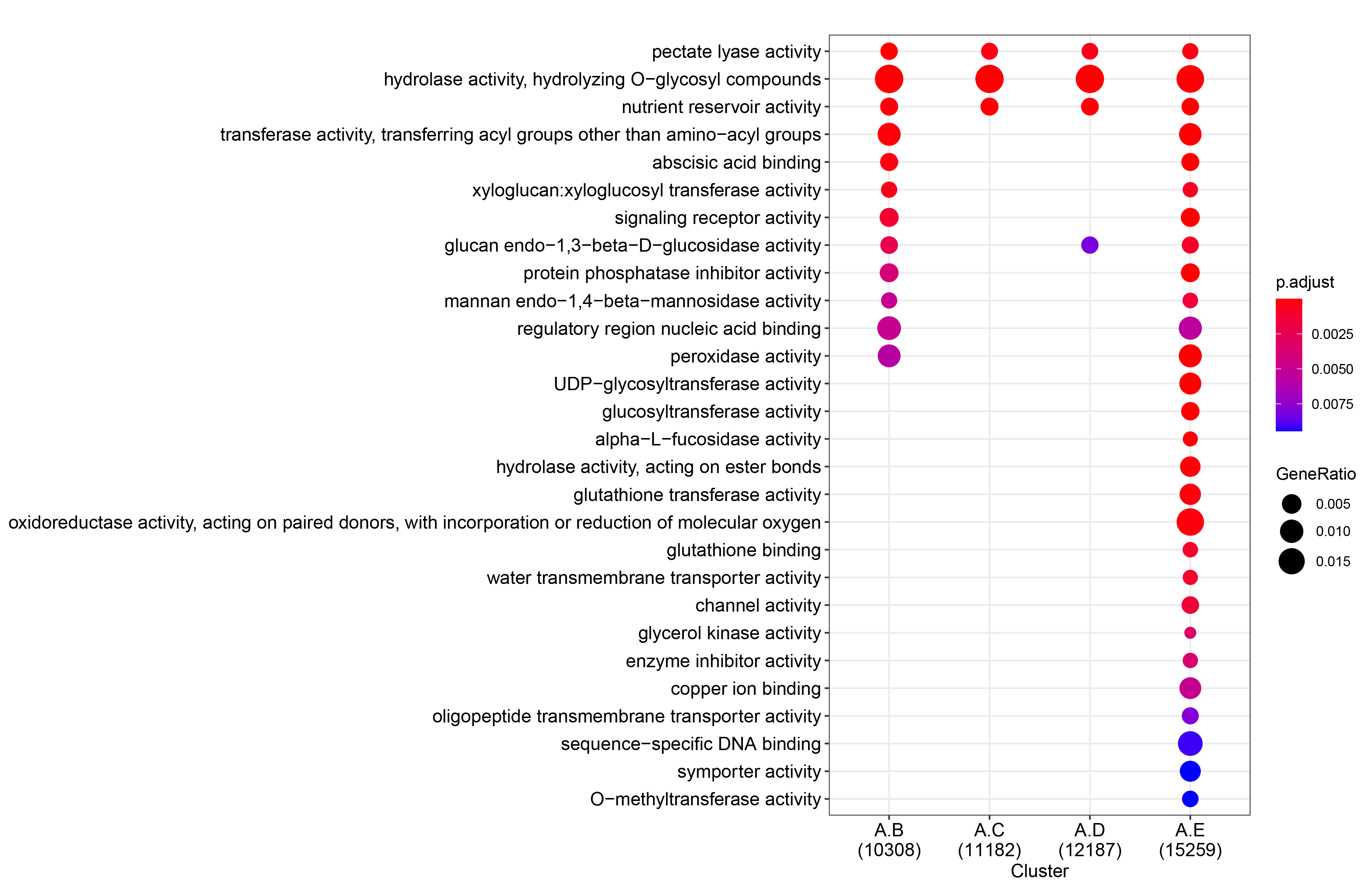

Supplement: Supplementary file 19 [file Image_5.jpeg]

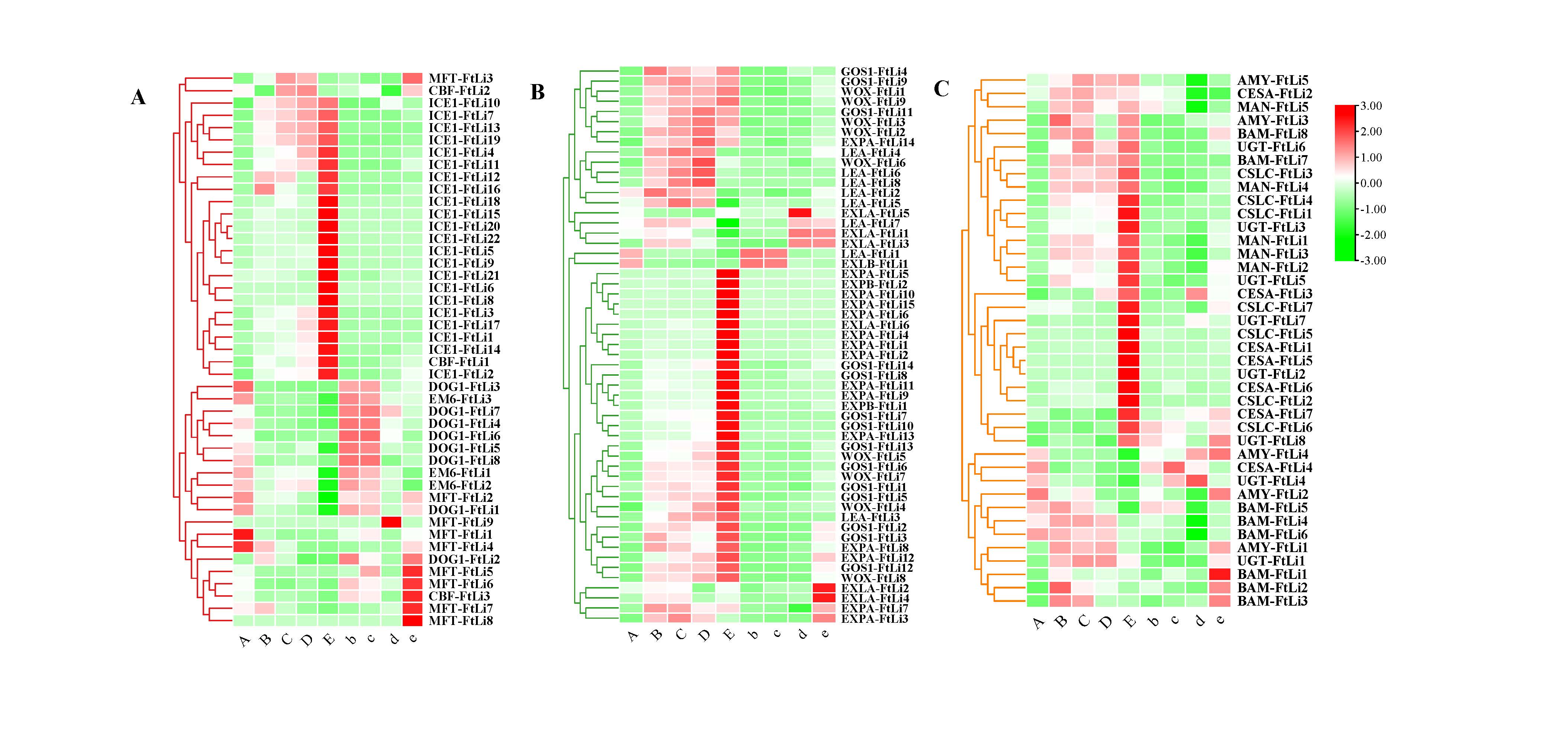

Supplement: Supplementary file 20 [file Image_6.jpeg]

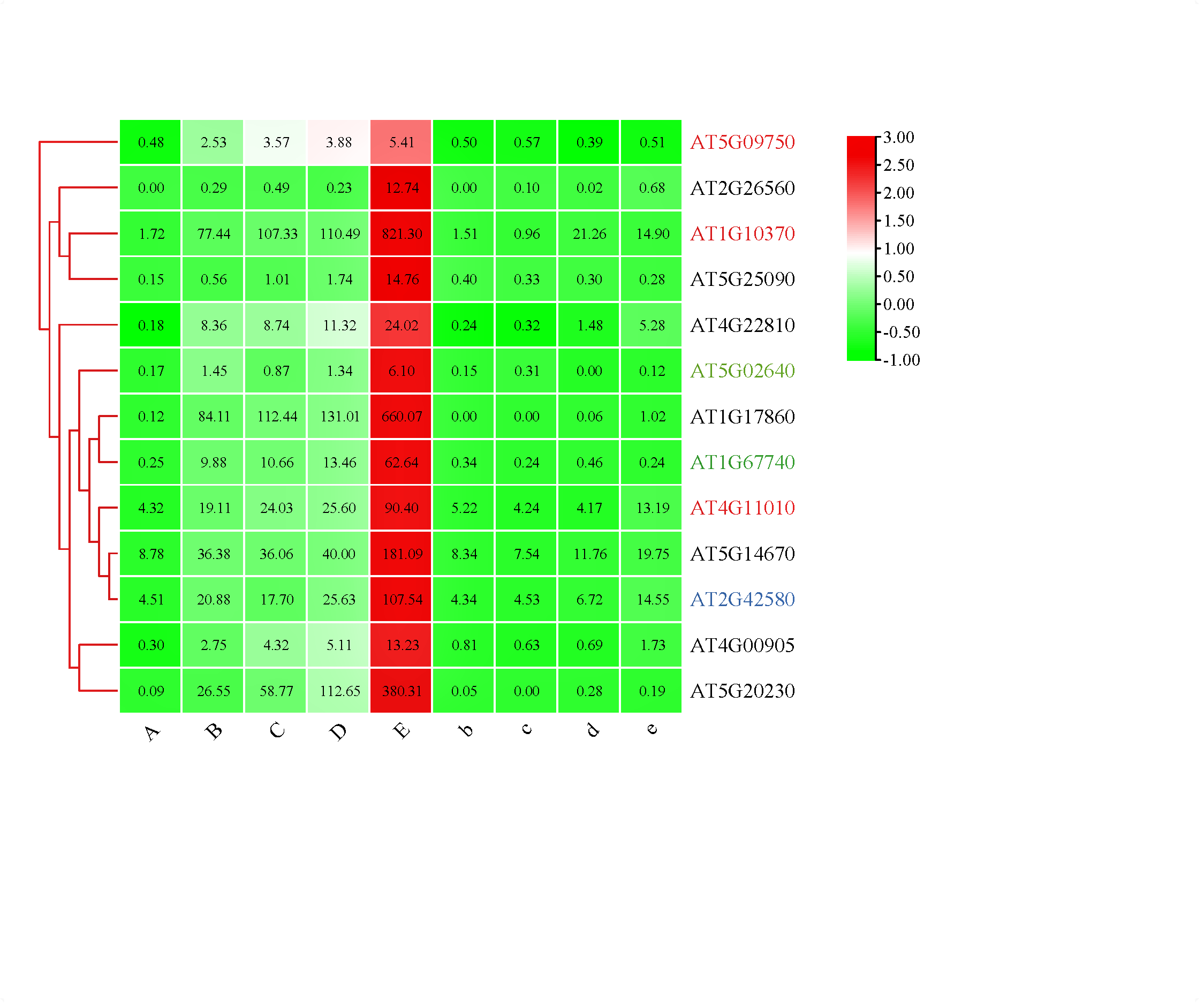

Supplement: Supplementary file 22 [file Image_8.tif]

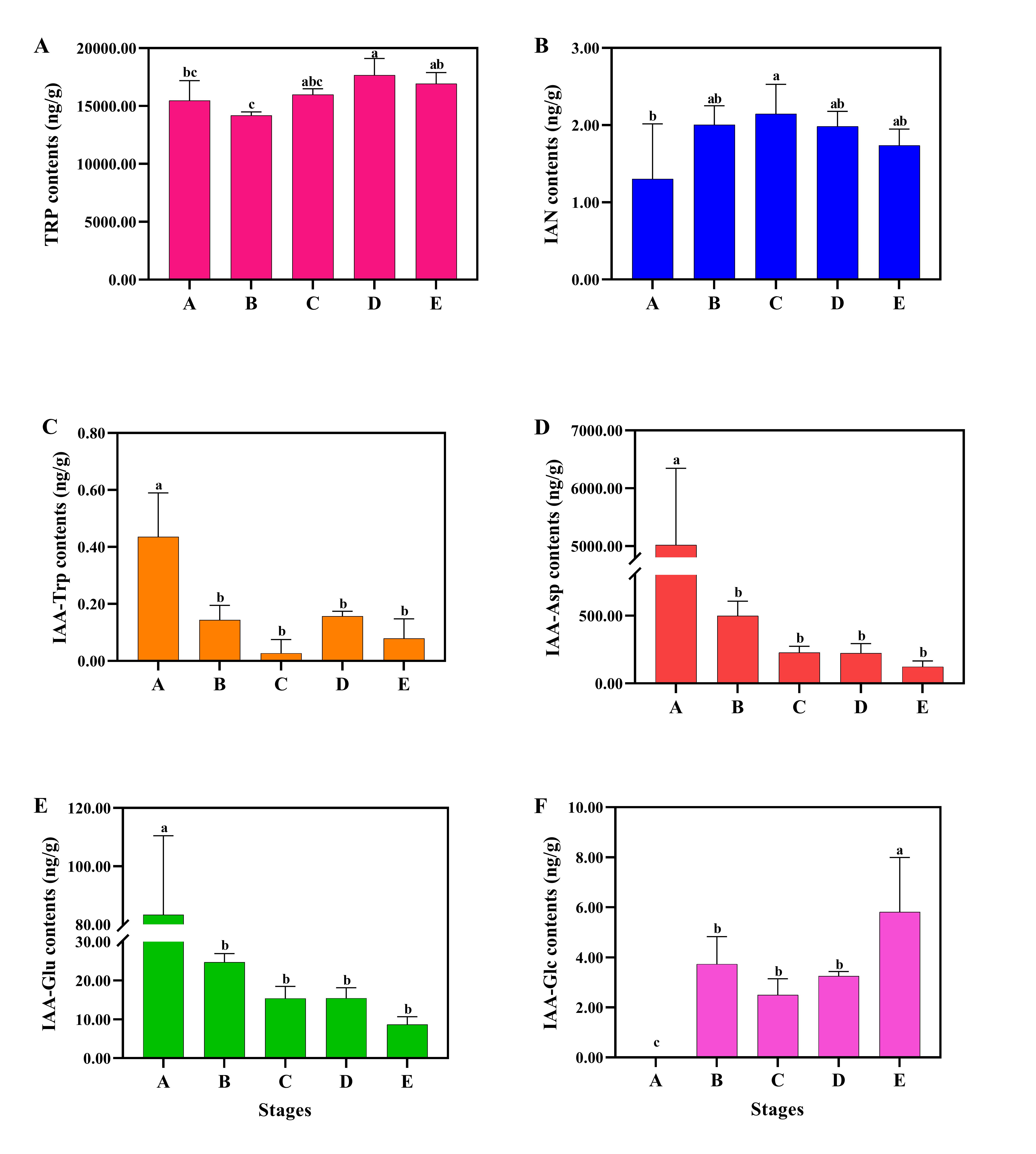

Supplement: Supplementary file 23 [file Image_9.jpeg]

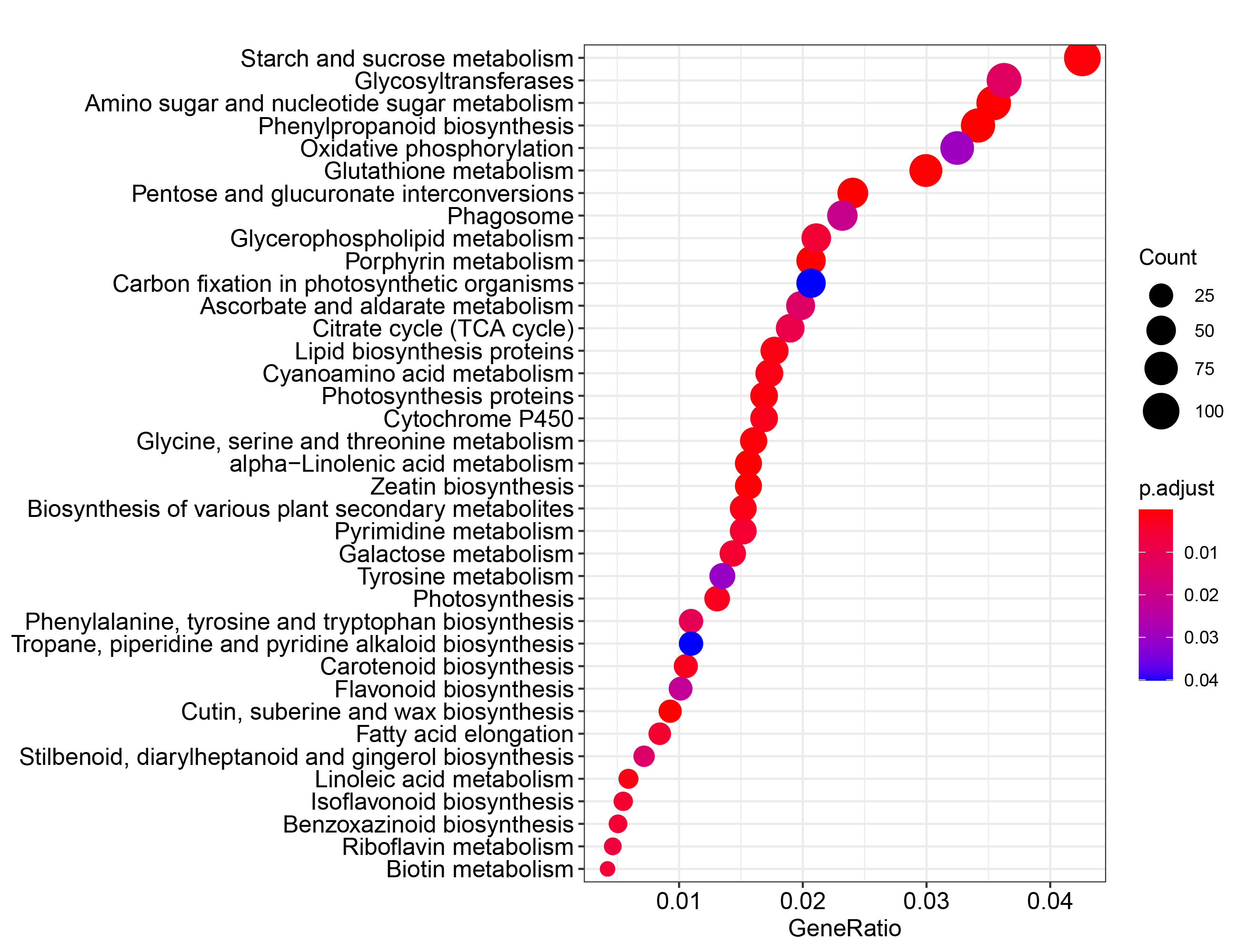

Supplement: Supplementary file 24 [file Image_10.jpg]
